# Remarks on Congestive Fever

**Published:** 1840-01-11

**Authors:** John R. Buck

**Affiliations:** Memphis, Tennessee


### REMARKS ON CONGESTIVE FEVER.

BY JOHN R. BUCK, M. D.

To the Editors of the Medical Examiner.

In No. 38, vol. 2d, of the Medical Examiner I reported a case of congestive fever, and made some remarks upon the nature of the disease, and the plan of treatment pursued in it in this part of the country. It is with no little satisfaction that I now record, in favour of my views, the testimony of my distinguished friend, Dr. N. L. Thomas, of Montgomery county, in this state, whose opinion upon any medical subject deserves the highest respect. Dr. T. writes to me as follows:

"After reading your case in the Examiner, I have determined to communicate some facts which may throw some light upon the question there started. James, æt. twelve, was taken with common intermittent fever in September. I ordered some pills of Carpenter's precipitated extract of bark,—four pills at night, and four the next morning,—to be given him. He came in and attended to some business in the house before breakfast, but was ordered back to the cabin, to be at rest. About 7 o'clock, his appearance attracted attention, and I went in to see him. I found him slightly delirious, with a small, frequent pulse, and great agitation of the nervous system, shown by spasmodic action of almost the whole muscular system. I concluded, after an examination, that it was the effect of the medicine, that it would subside soon, and left him to see a patient, but was soon sent for. When I got home, I found him pulseless and speechless. There being a kettle of water on the fire, I poured some of it, boiling, upon his legs, which made him cry out, and also speak, which he had refused to do before. I then placed a large sinapism over the epigastrium. His pulse became perceptible, and in some hours the irregular action of the muscles, and the delirium subsided, and next day he was able to resume his occupation.

The pills he had taken were prepared by Mr. T——, my student, (who was a novice in pharmacy,) and I was induced to weigh an equal number of them, which fell only two grains short of a drachm.

In my trials of the extract,—of which I have used several ounces,—I find it of about equal strength with quinine.

As far as this case goes, it proves that a powerful prostrating effect is produced by an overdose. I have no doubt but that a very little increase of its effect would have proved fatal. No other medicine was given, and there was no discharge, or anything else to account for the symptoms. It seems highly probable that the quinine in your case increased the prostrating effects of the chill.

I have bled several persons during the past summer, while labouring under a chill, and always with an improvement in the feelings of the patient, an elevation of the temperature, and a great alleviation of the other stages of the paroxysm; and what is best of all, there has been no relapse in any case, which you know uniformly occurs when checked by quinine."

This case adds another link to the chain of evidence already adduced against the stimulating plan of treatment in congestive fever, and goes far to establish the position I have assumed—that it is not a disease, " sui generis," but an aggravated, or malignant intermittent. The boy had common intermittent fever, evidently rendered congestive by an overdose of the extract of bark, given through mistake. The first effects of the medicine were powerfully stimulating—the whole nervous system was greatly agitated, "but all over excitement is followed by a corresponding depression," so, in a few hours afterwards he was both pulseless and speechless, requiring the most powerful revulsives to relieve the congestion.

The plan of treatment that I pursue in this disease will be better understood by giving the history of a case.

A negro man, about thirty years of age, was taken with a severe chill, which lasted about two hours. During the whole paroxysm he was slightly delirious. I saw him before his chill went entirely off. An expression of alarm was in his countenance,—a symptom always present at this stage of the disease. Pulse sixty-two, small and oppressed, but seemed to increase in force as it was pressed upon. Respiration twenty in the minute; eyes suffused; extremities cool. I opened a vein in the arm, but could get no blood, I then had his feet and legs put in hot water, and opened the temporal artery; it ran very slowly at first, but increased, until a pint had been taken, when it stopped. The patient said he felt infinitely better. His chill had entirely subsided; pulse was better; respiration easy; intellect clear, and skin perfectly natural. I ordered him to keep perfectly quiet, and left him. In about four hours I was again sent for. He was attempting to get out of his bed; delirium active; pulse eighty, full and strong; constantly calling for his tea. I should mention that, previous to my seeing him, he had drunk freely of a decoction of fodder, a remedy which he said had never failed to stop the chill in him before. I took from his arm (the same orifice which I had before opened) about twenty ounces of blood, as near as we could guess at it. He took a dose of sulphate of magnesia some hours afterwards, and the next day was convalescent.

Since writing the above, I have received the 45th number of the Examiner, containing "Remarks on the Malignant Intermittent and Remittent Fevers, of Limestone County, Alabama, by T. Stith Malone, M. D." These remarks, Dr. Malone says, were suggested particularly by my letter on congestive fever, above referred to. From the history of the disease, as given by him, there can be no doubt but that the malignant intermittent fever of that region, and the congestive fever of this, are one and the same disease. And his treatment proves very clearly, that it is not a disease of debility. When called to a patient, he opens a vein in each arm, and continues the abstraction of blood uutil the congestion is relieved, the chill is over, and a general diffusion of the energies, and an equalized circulation is established. If there be irritable stomach, or a sensation of weight or oppression there, he applies cups over the epigastrium.

So far the treatment of Dr. Malone is rational, and seems to be founded on a correct view of the pathology of the disease. All the indications seem to me to be fulfilled; the congestion is relieved, the chill is over, the circulation equalized, and the patient is at that point, when, in my experience, convalescence always commences. But Dr. Malone thinks differently. So soon as he has, by active and energetic depletion, restored the lost balance in the circulation, he endeavours to keep it restored by "active and energetic stimulation—throwing in large doses of quinine," from sixty to one hundred grains in six or eight hours. This mode of treatment appears to me inconsistent. If blood-letting will restore the balance, why will it not keep it restored? There is no necessity of carrying it to the extent, sufficient to produce prostration, and if quinine is required at all, give it in moderation. The vital energies are only sustained by the action of stimuli upon excitability, and if there be not excitability enough to sustain the system at the period to which it is stimulated, a dangerous degree of depression will inevitably follow. If, when by action and energetic depletion, the congestion is relieved, the circulation equalized, and the patient "quieted," he were allowed to remain so, I venture the assertion that the cases of relapse, of which Dr. Malone speaks, would be less frequent, and the harrassing effects of salivation, which seem indispensable in such cases, would be avoided.

*Memphis, Tennessee, Dec. 26th,* 1839.

